# A Colorimetric Approach for Detecting a Selected Fecal Cortisol Metabolite as a Stress Biomarker in Atlantic Salmon

**DOI:** 10.1021/acsomega.4c10562

**Published:** 2025-06-13

**Authors:** Ernestine Fanjara, Reidar Arneberg, Olav M. Kvalheim, Yanran Cao, Grete K. F. H. Aas, Vera Kristinova, Asgeir Sæbø, Anne Stene

**Affiliations:** † Department of Biological Sciences Aalesund, 8018 Norwegian University of Science and Technology, NTNU in Aalesund, P.O. Box 1517, Aalesund NO-6025, Norway; ‡ Innolipid AS, Tonningsgate 17, NO-6006 Aalesund, Norway; § Department of Chemistry, 1658 University of Bergen, P.O. Box 7803, Bergen NO-5020, Norway

## Abstract

Atlantic salmon (Salmo salar) is a key aquaculture species in Norway, where maintaining optimal welfare and minimizing stress are crucial for sustainable production. Cortisol and its metabolites are widely recognized as reliable biomarkers for fish stress assessment. Although traditional cortisol measurement methods are effective, they typically require complex, labor-intensive laboratory procedures, limiting their feasibility for on-site aquaculture use. Conversely, colorimetry-based ultraviolet–visible (UV–vis) spectrophotometry presents a rapid and cost-effective alternative, yet its application for monitoring stress in fish remains underexplored. Therefore, this study aimed to develop a simple, rapid, and cost-effective colorimetric assay for detecting fecal tetrahydrocortisone (5β-THE), the predominant cortisol metabolite in Atlantic salmon feces, following the establishment of its baseline level under standard farming conditions. For this purpose, fecal samples collected over six months were analyzed using a validated LC–MS/MS method, revealing consistently low 5β-THE levels in nonstressed fish (average 533.81 ± 66.7 ng/g). Additionally, a blue-tetrazolium-based UV–vis spectrophotometric assay was developed for detecting 5β-THE. Key parameters, including solvent systems and reagent concentrations, were systematically optimized. The method exhibited excellent linearity (*R* ^2^ = 0.997) and satisfactory detection limits (394.75 ng/mL for methyl *tert*-butyl ether and 498.54 ng/mL for tert-amyl alcohol systems). Although these detection limits were higher than expected in hydrolyzed fecal samples, the findings suggest the potential of this method for rapid, on-site stress assessment using raw samples, as 5β-THE concentrations in such samples exceeded the detection threshold. Future research will focus on optimizing the sample preparation process and validating the method.

## Graphical abstract

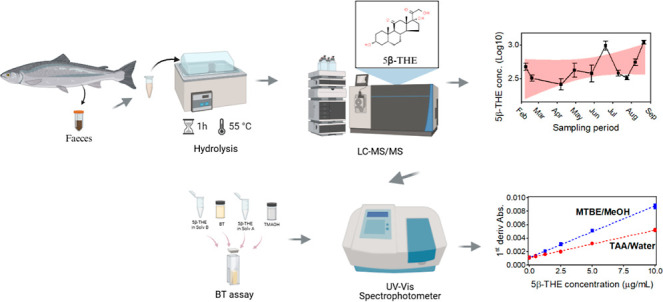

## 1 Introduction

Being one of the major, high-value finfish species produced in aquaculture worldwide, Atlantic salmon (Salmo salar) is of extensive commercial importance.^1–3^ To further enhance the production and profit margin while simultaneously aiming for sustainability and reducing negative interactions with the surrounding environment, fish feed composition, management of farming operations, and the cage environment are among the areas that can be optimized.^4,5^ Studies have shown that suboptimal conditions and stress may lead to poor fish health and welfare, increased mortality, and a negative reputation of the industry.^6^ User-friendly tools are crucial to understanding the fish’s response to events in its environment. Understanding the stress responses is essential for optimizing aquaculture practices and ensuring animal welfare in aquaculture. Identifying fish stress responses can improve specific farming technology and operations by comparing alternative solutions to existing pumps, delousing, and net cleaning as well as identifying latent infections and adverse conditions in the natural marine environment. Cortisol, a steroid hormone released in response to stress, is widely recognized as a primary biomarker for assessing stress levels in fish and other vertebrates.^7–9^ Elevated cortisol levels are often a response to stressful events but also poor water quality, visual and auditory disturbances, and the onset of diseases.^10,11^

Cortisol levels in fish have been measured using different techniques such as radio-immunoassays,^12–15^ chemiluminescent assays,^16^ enzyme-linked immunosorbent assays (ELISA),^17–20^ high-performance liquid chromatography (HPLC),^15,21^ and liquid chromatography-tandem mass spectrometry (LC–MS/MS).^22,23^ While these techniques are effective in measuring changing levels of cortisol, they often involve complex and time-consuming procedures, requiring specialized and space-requiring equipment as well as expertise.^24,25^ Offering a more accessible approach for quick assessment of stress, ultraviolet–visible (UV–vis) spectrophotometry has re-emerged as a promising alternative for cortisol analysis. It has recently gained attention for its potential to simplify cortisol detection in clinical studies.^25–29^ Its principle is based on a substance’s ultraviolet or visible light absorption. According to Beer–Lambert’s law, the absorbance, typically measured in the range of 200–800 nm, is directly proportional to the concentration of the analyte.^30^

Recent advancements in UV–vis spectrophotometry have focused on improving its sensitivity and specificity for cortisol detection. Researchers have developed novel assays and investigated reagents interacting specifically with cortisol and enabling the measurement of cortisol levels with high accuracy and precision.^26,31^ For instance, a study conducted by Ahmed et al.^27,32^ explored the use of colorimetry-based UV–vis spectrophotometry for optical sensing of human salivary cortisol, demonstrating its potential for real-time measurement and stress monitoring. The study highlighted the technique’s efficacy in fingerprinting stress-related biochemical changes, offering a reliable alternative to traditional, more invasive methods of cortisol assessment. In other studies, UV–vis spectrophotometry has been integrated with innovative materials and technologies such as sensors to enhance its analytical capabilities and detection limits, allowing for more sensitive cortisol measurements even at low concentrations.^33,34^

While sensor-based UV–vis techniques^34,35^ (or sensors combined with amperometry^36^) offer high sensitivity and specificity, they are less commercially available and require specialized expertise for sensor development. In contrast, colorimetric-based analysis is straightforward and easy to implement. Its rapid analysis time, low cost, and minimal sample preparation make it well suited for field studies and real-time monitoring in aquaculture environments. These attributes not only enhance farmers’ and veterinarians’ ability to monitor cortisol levels during stressful events but also provide valuable insights into fish physiological responses to various environmental conditions and aquaculture practices, potentially leading to their improvement. Yet, despite growing interest and advancements in medical research, the application of colorimetric methods, and UV–vis-based techniques in general, for measuring cortisol in fish remains relatively rare.^35^

In Atlantic salmon, tetrahydrocortisone (5β-THE) was previously found to be the most predominant cortisol metabolite in feces and suited as a reliable, low-invasive stress biomarker for this fish species.^23,37^ Feces samples, as opposed to blood, can be collected less invasively from fish through gentle stripping. Based on this knowledge, this study was conducted to test the relevance and effectiveness of colorimetry-based UV–vis spectrophotometry as a rapid and low-cost method for measuring 5β-THE levels in Atlantic salmon feces. This was achieved through:

(1) establishing the baseline level of fecal 5β-THE from fish reared under presumably normal farming conditions. Fish feces were collected over six months and analyzed with a validated LC–MS/MS method.
(2) developing a rapid, simple, and low-cost Blue tetrazolium (BT)/UV–vis method in an attempt to simplify the measurement procedure. Different factors and experimental conditions were considered for the optimization of the method. These included different solvent systems and varying concentrations of reagent and catalyst.

## 2 Results

### 2.1 Farming Site and Fish Population

At the aquaculture production area where the farming site is located, it is required by Norwegian laws and regulations that the level of mature female sea lice should not exceed 0.2 from the beginning of week 16 to the end of week 21 (18 April to 29 May 2022 in this case) and 0.5 for the other periods.^38,39^ As shown in Figure 1a, the number of female lice per fish in the farming site was below the national threshold during the study period. Even sea lice levels reaching the national threshold rarely harm farmed fish, but they are set to protect wild salmonids. The significant falls in female lice count in Figure 1a correspond to the delousing activities performed at the farming site during the study period. Part of the farming site was subjected to delousing on April 24, 2022, and the entire site on June 24, 2022, and July 29, 2022, respectively. The fish used in the present study were deloused on June 24 and July 29 but not on April 24, 2022.

**Figure 1:**
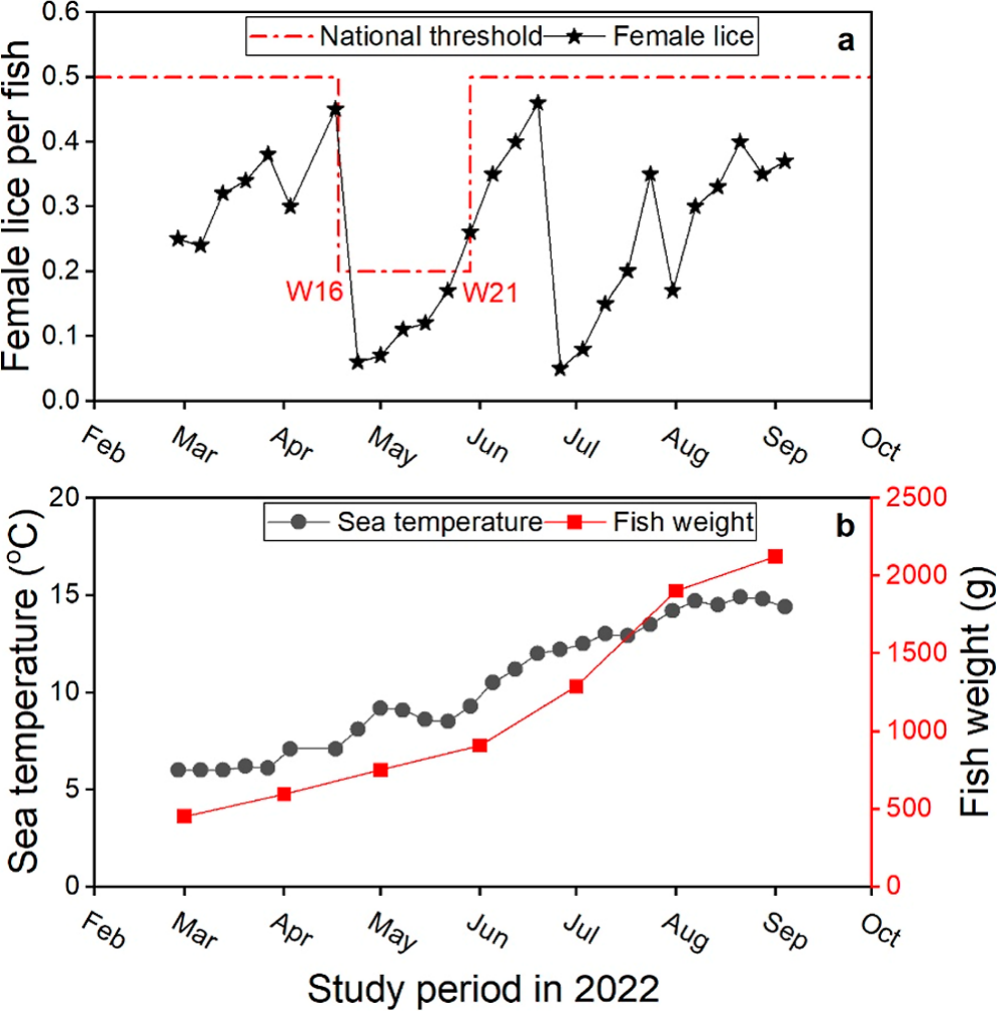
Sea lice count (a), sea temperature (b, black), and fish weight (b, red) recorded at the farming site during the sampling period. The red line in Figure 1a shows the national threshold for sea lice numbers. The limit is 0.2 between week 16 (W16) and week 21 (W21) and 0.5 otherwise.

As shown in Figure 1b, the sea surface temperature at the farming site increased toward the summer/early autumn, from 6 °C in February to reach a top value of 14.9 °C in August (Figure 1b). It then started to flatten by the end of the study period. According to the farmers, no problems with wounds or diseases were observed during the sampling period from February to September. The fish exhibited normal growth, increasing from an average body weight of 453 g at the start of the study period to 2125 g at the end (Figure 1b).

### 2.2 Fish Stress Level Measured by LC–MS/MS

The 5β-THE levels observed throughout the study period were comparable to those previously reported for nonstressed fish,^37^ except for fish sampled on 3 July and 4 September 2022 (Figure 2). After removing these two cases considered as outliers, the mean fecal 5β-THE level for nonstressed fish was 533.81 (±66.74) ng/g, with a median of 384.69 [304.31, 557.39] ng/g. A trend of increasing 5β-THE levels was observed for the later study period (after the second delousing event). It reached a significantly higher value of 1113.23 [998.52, 1301.77] ng/g at the last sampling date of 4 September 2022, compared to the control group 542.69 [410.48, 682.72] ng/g (*p* = 0.01). This is also the period with the highest recorded sea temperatures, as shown in Figure 1b (August–September).

**Figure 2:**
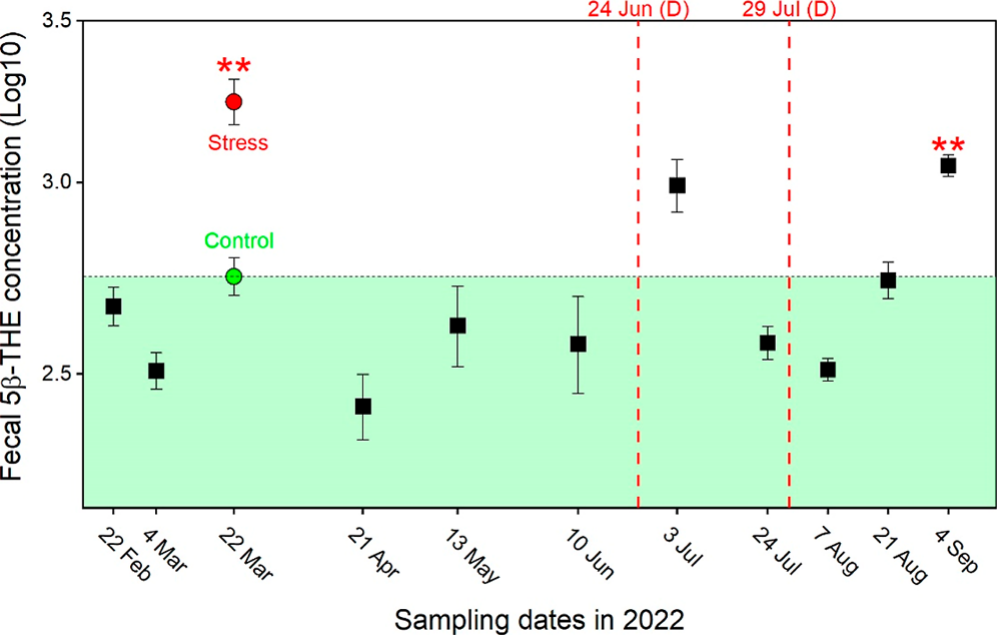
Fecal 5β-THE concentrations measured in fish during normal farming conditions (black). Each data point represents the mean (±SEM) of Log10-transformed values from 8 to 10 fish. Data for the control group (green) and stress group (red) were obtained from the study of Fanjara et al.,^37^ which were measured by LC–MS/MS and served as reference values. The green area indicates a zone of nonstressed level. The asterisk represents 5β-THE values statistically significantly higher than the control group’s. The studied fish were subjected to delousing (D) on 24 June and 29 July 2022, respectively.

### 2.3 Colorimetric Assay

Based on the results obtained from the LC–MS/MS analyses presented above, the 5β-THE level in raw feces of nonstressed fish reared under normal farming conditions is, on average, 533.81 (±66.74) ng/g. The colorimetric method was developed and optimized to detect at least this concentration. Furthermore, it was considered that the concentration to be measured was lower (around 26.69 ng/g, dilution factor 20) in dried methyl *tert*-butyl ether (MTBE) extract after hydrolysis.

In a preliminary trial, the method described by Ahmed^32^ and Tu et al.^26^ for colorimetric measurement of cortisol in human saliva and sweat was used to measure methanolic solutions of cortisol and 5β-THE. However, no color development was observed within the specified reaction time of 10 min or even overnight. Hence, an optimization process was conducted, as described in Section 5.3.3.

#### 2.3.1 MTBE with Methanol (MTBE/MeOH)

The experimental conditions tested and the results obtained from Design 1 are presented in Table 1.

**Table 1:** MTBE/MeOH Design 1 Layout (2^3^ Factorial with 3 Center Points) and Obtained Results

	blue tetrazolium (BT), mg/mL	tetramethylammonium hydroxide (TMAOH),%	methanol (MeOH), %	first deriv. of *A*_max_ ^a^	Time to *A*_max_, min^b^
Exp 1	5	1	15	0.00821	20
Exp 2	10	1	15	0.00855	18
Exp 3	5	5	15	0.00843	4
Exp 4	10	5	15	0.00853	5
Exp 5	5	1	35	0.00732	20
Exp 6	10	1	35	0.00744	20
Exp 7	5	5	35	0.00876	12
Exp 8	10	5	35	0.00868	11
Cent 1	7.5	3	25	0.00865	11
Cent 2	7.5	3	25	0.00803	11
Cent 3	7.5	3	25	0.00875	12

a Maximum absorbance (*A*_max_) in the first derivative form.

b Time to reach *A*_max_ in min.

The experiments were performed in random order with one center point at the beginning, one in the middle, and one at the end of the series. Results from regression analysis were used to identify the significance of each of the predictor variables (BT, tetramethylammonium hydroxide (TMAOH), and percentage MeOH) and their interactions, using Lenth’s margin of error (ME) and simultaneous ME (SME).^40^ The results from Design 1 reveal that none of the factors strongly affected the absorbance. Their absolute regression coefficient values were below Lenth’s SME of 1.43 (Figure 3a). TMAOH and its interaction with MeOH were within the zone of uncertainty, with absolute regression coefficient values falling between SME (1.43) and ME (0.42). The remaining two variables were nonsignificant, with MeOH (very close to the ME limit) having a higher effect than BT (Figure 3a). Looking only at the main effects in the regression plot, TMAOH and MeOH depicted opposite effects on absorbance. Increasing TMAOH improved the absorbance reading, while increasing methanol content decreased it. A closer look at the contour plot (Figure 3c) shows, however, that an increase in MeOH reduces the observed absorbance at low TMAOH values (1%), while the effect of increasing MeOH is opposite at high TMAOH values (5%). This means that MeOH’s effect strongly depends on the amount of TMAOH.

**Figure 3:**
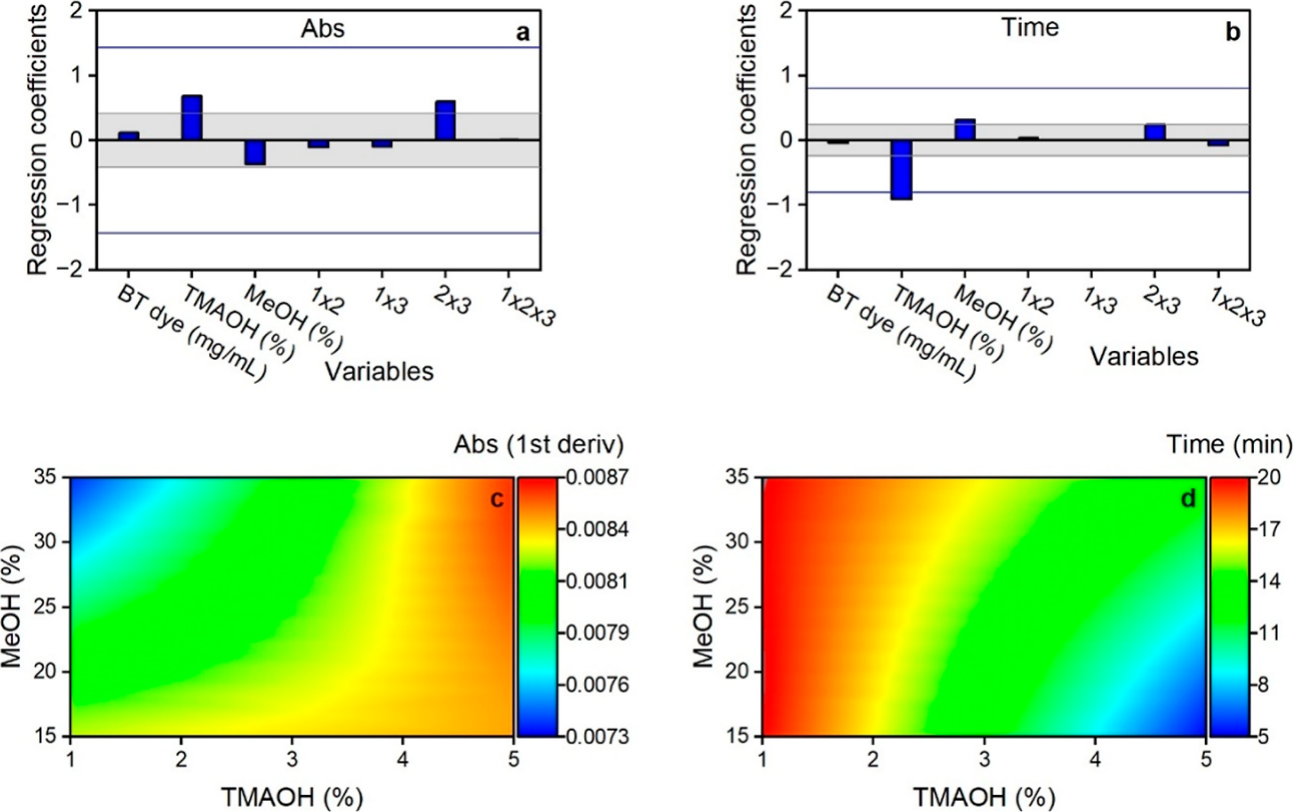
Effects of BT, TMAOH, and MeOH on sample absorbance (a,c) and time to reach maximum absorbance (b,d) based on the conditions used in MTBE/MeOH Design 1. The gray area between Lenth’s ME lines in the regression plots (a,b) is a region of nonsignificance. The blue lines represent Lenth’s SME. Factors with regression coefficients exceeding the SME lines are considered significant.

For the time needed to reach maximum absorbance, TMAOH was a significant factor. The absolute value of its regression coefficient exceeded the SME limit of 0.8 (Figure 3b). MeOH, on the other hand, was on the borderline of being significant, and BT was nonsignificant (regression coefficient below the ME value of 0.24). Based only on the main effects, TMAOH and MeOH also had opposite effects on time but the inverse of their effects on absorbance. The contour plot in Figure 3d shows that when TMAOH was increased, the time to reach maximum absorbance decreased for the whole interval of values of MeOH. The effect of MeOH was, however, more significant at high values of TMAOH (5%) than at low values. Increasing the methanol concentration increased the time to reach maximum absorbance (color development was delayed).

Based on the results from Design 1, we could limit the variation regions for the three selected variables to reach maximum absorbance within a preferred time region of 10 to 15 min. As we can see in Figure 3c and d, high TMAOH (>3%) and medium range of MeOH (20–30%) yielded high absorbance and acceptable time for reaching this maximum value (10 to 15 min). As a result, the TMAOH concentration range was increased to 3–7%, and the MeOH percentage range narrowed to 20–30% in the next design. The BT concentration range was kept unchanged as this variable was not important. Table 2 summarizes the experimental conditions tested in Design 2, as well as the maximum absorbance and the time to reach the maximum absorbance obtained from this design.

**Table 2:** MTBE/MeOH Design 2 Layout (2^3^ Factorial and 3 Center Points) and Obtained Results

	BT, mg/mL	TMAOH, %	MeOH, %	first deriv. of *A*_max_ ^a^	time to *A*_max_, min^b^
Exp 1	5	3	20	0.00895	12
Exp 2	10	3	20	0.0083	9
Exp 3	5	7	20	0.00854	5
Exp 4	10	7	20	0.00817	4
Exp 5	5	3	30	0.00855	18
Exp 6	10	3	30	0.00846	16
Exp 7	5	7	30	0.00851	9
Exp 8	10	7	30	0.00846	7
Cent 1	7.5	5	25	0.00837	8
Cent 2	7.5	5	25	0.00844	8
Cent 3	7.5	5	25	0.00866	9

a Maximum absorbance (*A*_max_) in the first derivative form.

b Time to reach *A*_max_ in min.

In Design 2, none of the variables were significant for absorbance. The absolute values of their regression coefficients were all below the SME (3.99) and ME (1.18) limits. However, unlike in Design 1, BT had a stronger effect on absorbance than TMAOH and MeOH (Figure 4a). Further increasing TMAOH in this design did not improve the absorbance reading but instead decreased it, especially at low BT values of 5 mg/mL (Figure 4c). Similarly, although not significant, a higher BT concentration decreased the absorbance obtained at all TMAOH levels (Figure 4c).

**Figure 4:**
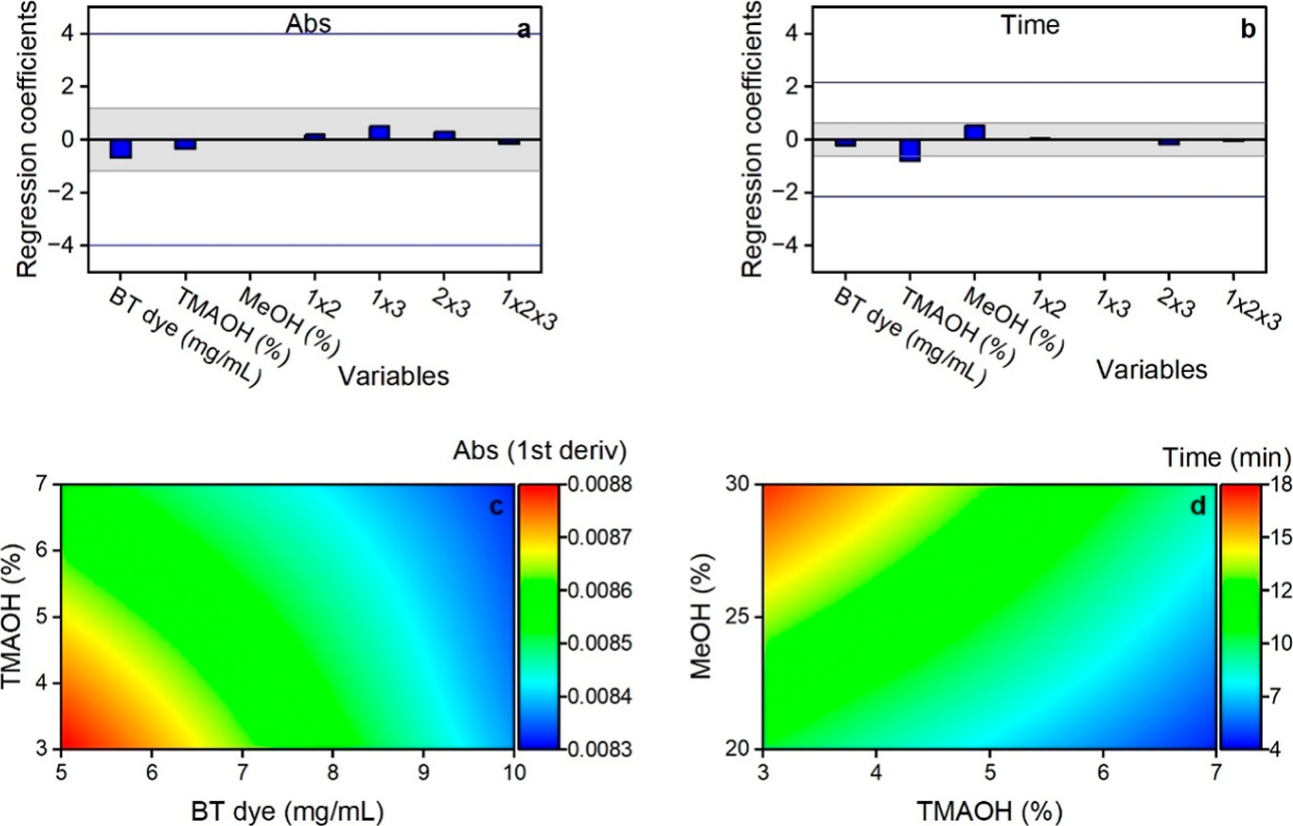
Effects of BT, TMAOH, and MeOH on sample absorbance (a,c) and time to reach maximum absorbance (b,d) based on MTBE/MeOH Design 2. The gray area between Lenth’s ME lines in the regression plots (a,b) is a region of nonsignificance. The blue lines represent Lenth’s SME. Factors with regression coefficients exceeding the SME lines are considered significant.

The effects of the three variables on time in Design 2 were similar to those observed in Design 1, although none of the factors were significant (regression coefficients below an SME of 2.16). TMAOH had the highest effect, followed by MeOH and BT, respectively (Figure 4b). Increasing TMAOH accelerated color development, while increasing MeOH delayed it (Figure 4d).

Since no significant variable was observed in Design 2, we concluded that the regions for variable variation are acceptable for reaching the maximum absorbance. In addition to high absorbance and the appropriate length of reaction time, it was also a prerequisite that the maximum absorbance obtained was stable over time to obtain robust measurements of absorbance. Slight deviations in incubation and acquisition times should not affect the read absorbance. When absorbance was plotted against time (Figure 5), the center points from Design 2 were found to fulfill these requirements (Figure 5e). Hence, medium amounts of MeOH (25%), BT (7.5 mg/mL), and TMAOH (5%) from Design 2 were chosen for the final method. As shown in Figure 5e, these experimental conditions resulted in an acceptable time for reaching the maximum absorbance (about 8 min), which was stable for at least 10 min.

**Figure 5:**
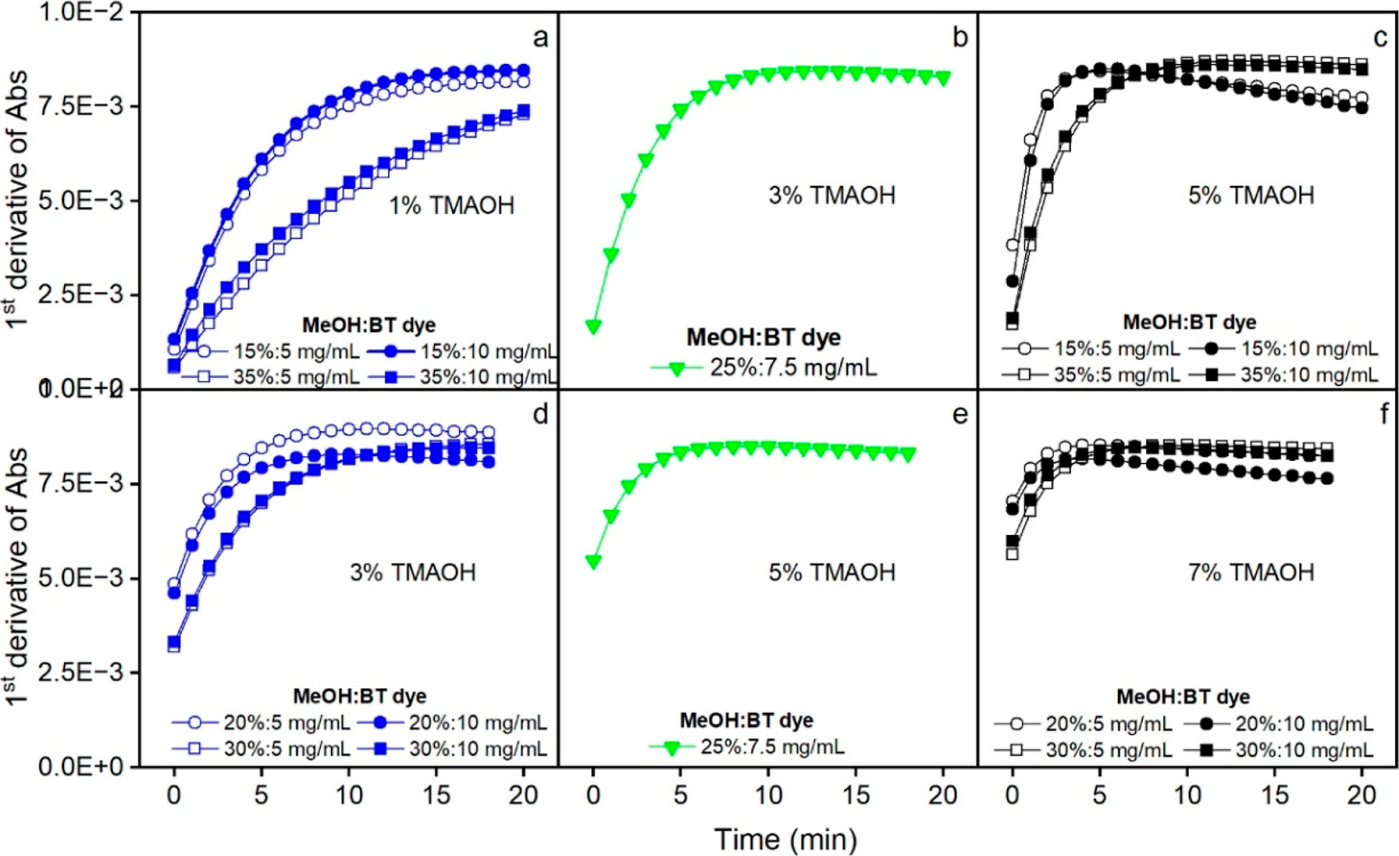
Changes over time of the absorbance values measured for 5β-THE (10 000 ng/mL) using the MTBE/MeOH system (a–f).

#### 2.3.2 *tert*-Amyl Alcohol with Water (TAA/Water)

Table 3 outlines the experimental conditions tested for Design 1 of the *tert*-amyl alcohol (TAA)/water system and the results obtained.

**Table 3:** TAA/Water Design 1 Layout (2^3^ Factorial and 3 Center Points) and the Obtained Results

	BT, mg/mL	TMAOH, %	water, %	first deriv. of *A*_max_ ^a^	time to *A*_max_, min^b^
Exp 1	5	3	40	0.00566	8
Exp 2	10	3	40	0.00482	6
Exp 3	5	7	40	0.00543	3
Exp 4	10	7	40	0.00511	2
Exp 5	5	3	60	0.00148	12
Exp 6	10	3	60	0.0016	8
Exp 7	5	7	60	0.00079	6
Exp 8	10	7	60	0.0016	7
Cent 1	7.5	5	50	0.00358	6
Cent 2	7.5	5	50	0.00299	5
Cent 3	7.5	5	50	0.00282	5

a Maximum absorbance (*A*_max_) in the first derivative form.

b Time to reach *A*_max_ in min.

In this design, the water content was the only significant factor observed to affect absorbance (Figure 6a). Its coefficient value exceeded Lenth’s SME of 0.64. The lower the water content, the higher the absorbance measured (Figure 6c). Conversely, the time to reach maximum absorbance was only slightly affected by both the water content and TMAOH concentration. However, none of these factors were significant (Figure 6b). The regression coefficients were below Lenth’s SME and ME of 3.48 and 1.02, respectively. A longer time was needed to reach maximum absorbance at high water content and low TMAOH percentages (Figure 6d). High TMAOH concentration resulted in a reaction that was too fast, with the absorbance value being less stable over time around maximum absorbance.

**Figure 6:**
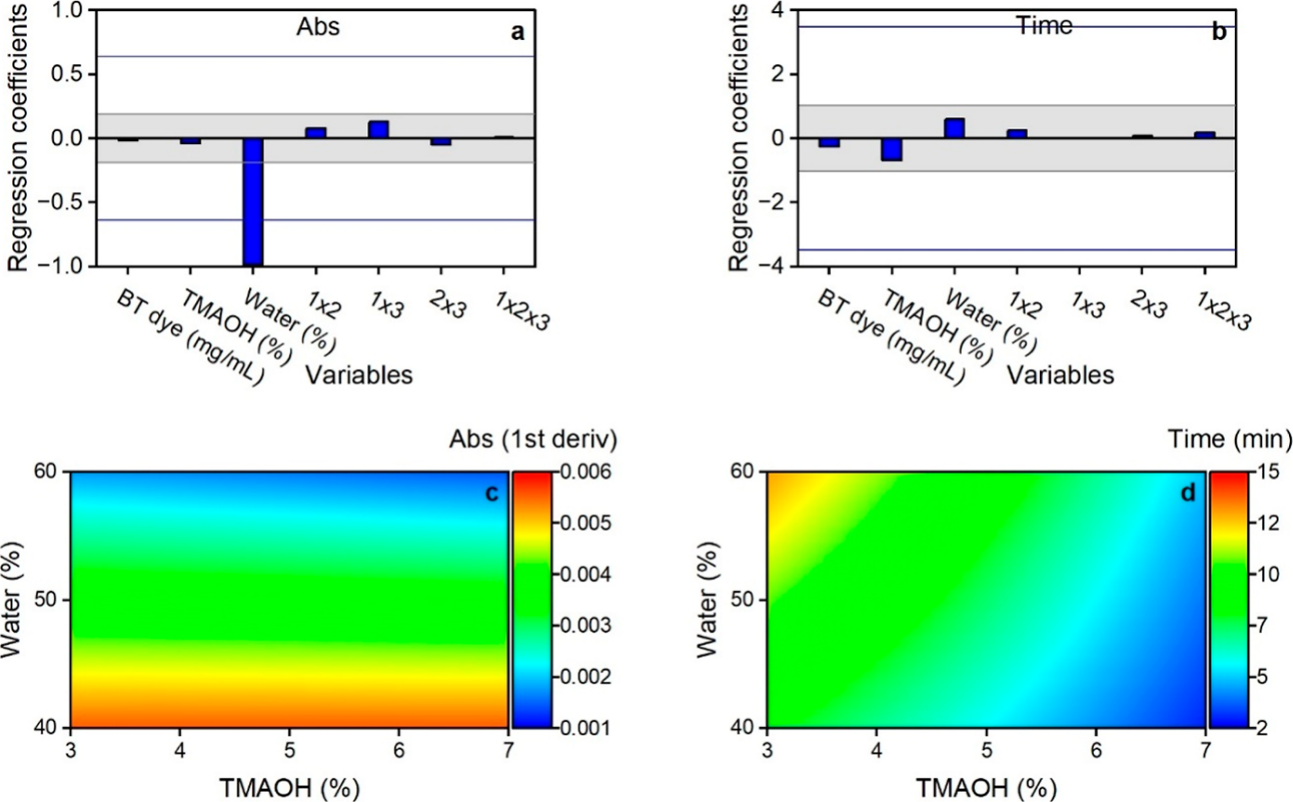
Effects of BT, TMAOH, and water on sample absorbance (a,c) and time to reach maximum absorbance (b,d) based on the experimental conditions in TAA/water Design 1. The gray area between Lenth’s ME lines in the regression plots (a,b) is a region of nonsignificance. The blue lines represent Lenth’s SME. Factors with regression coefficients exceeding the SME lines are considered significant.

Design 1 shows that the percentage of water is significant. A lower percentage of water will increase the absorbance and keep the time within acceptable values (at low TMAOH levels). Based on this information, a second design was created, with the water content reduced to 20–40%. The region used for the variable TMAOH was also reduced to 2–4%.

However, none of the factors were significant for absorbance (results not shown).

Considering the requirement for stable absorbance over time, the experimental conditions with 40% water used in Design 1 were selected for the final method (Figure 7c). It comprised 5 mg/mL BT, 3% TMAOH, and 40% water. This resulted in an acceptable time to reach the maximum absorbance (8 min), which was stable for at least 10 min.

**Figure 7:**
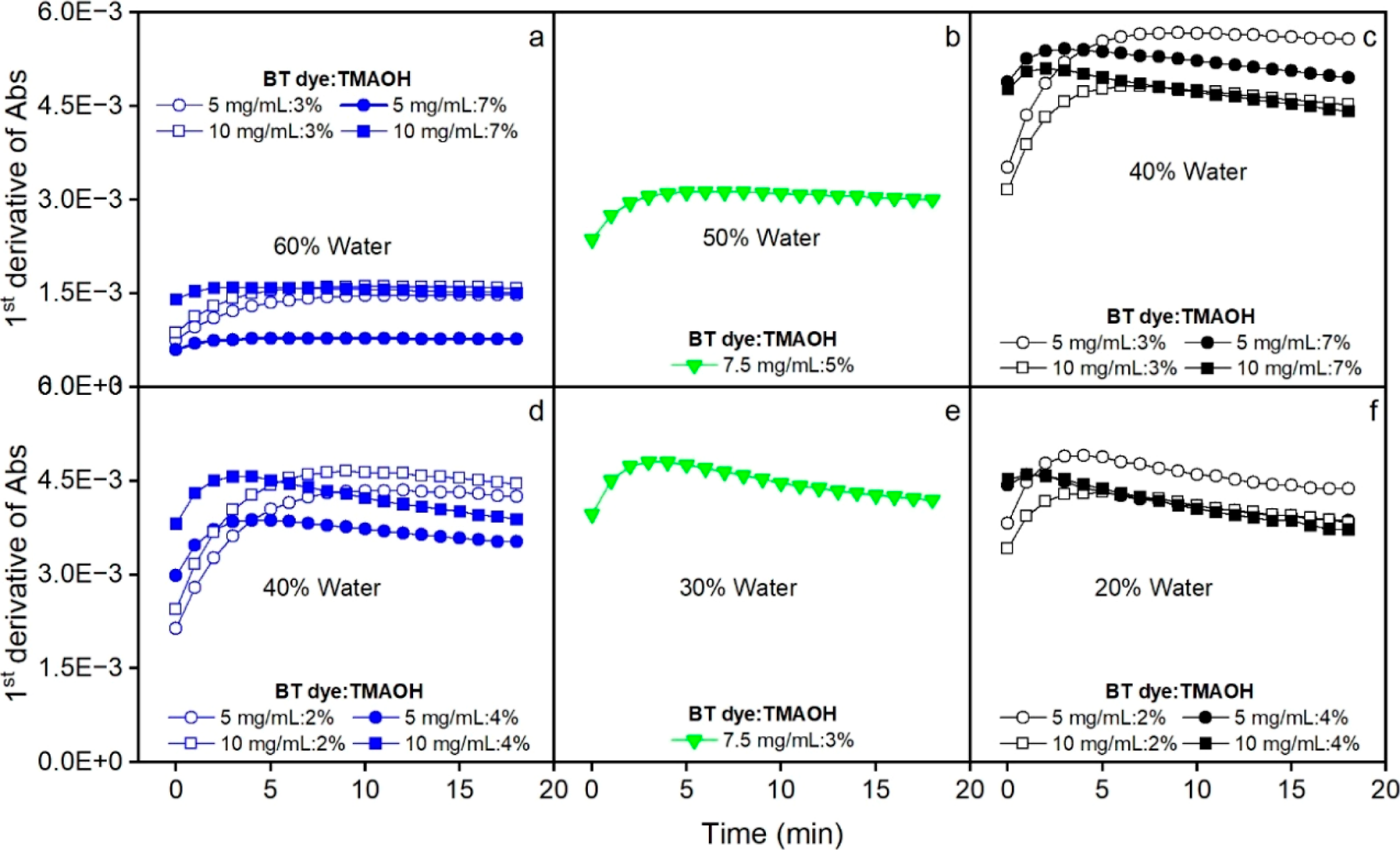
Temporal changes of the absorbance values measured for 10 000 ng/mL 5β-THE standard solution using the TAA/water system (a–f).

#### 2.3.3 Final Colorimetric Methods and Fecal 5β-THE Analysis

Using the conditions determined above, a decreasing concentration of 5β-THE was measured to determine the lowest concentrations detectable by the methods. After smoothing and differentiation (first-order derivative) of the spectral data (Figure 8), the absorbance value calculated for each level was plotted against the 5β-THE concentration (Figure 8c,f). Excellent linearity was obtained for both methods (*R* ^2^ = 0.997).

**Figure 8:**
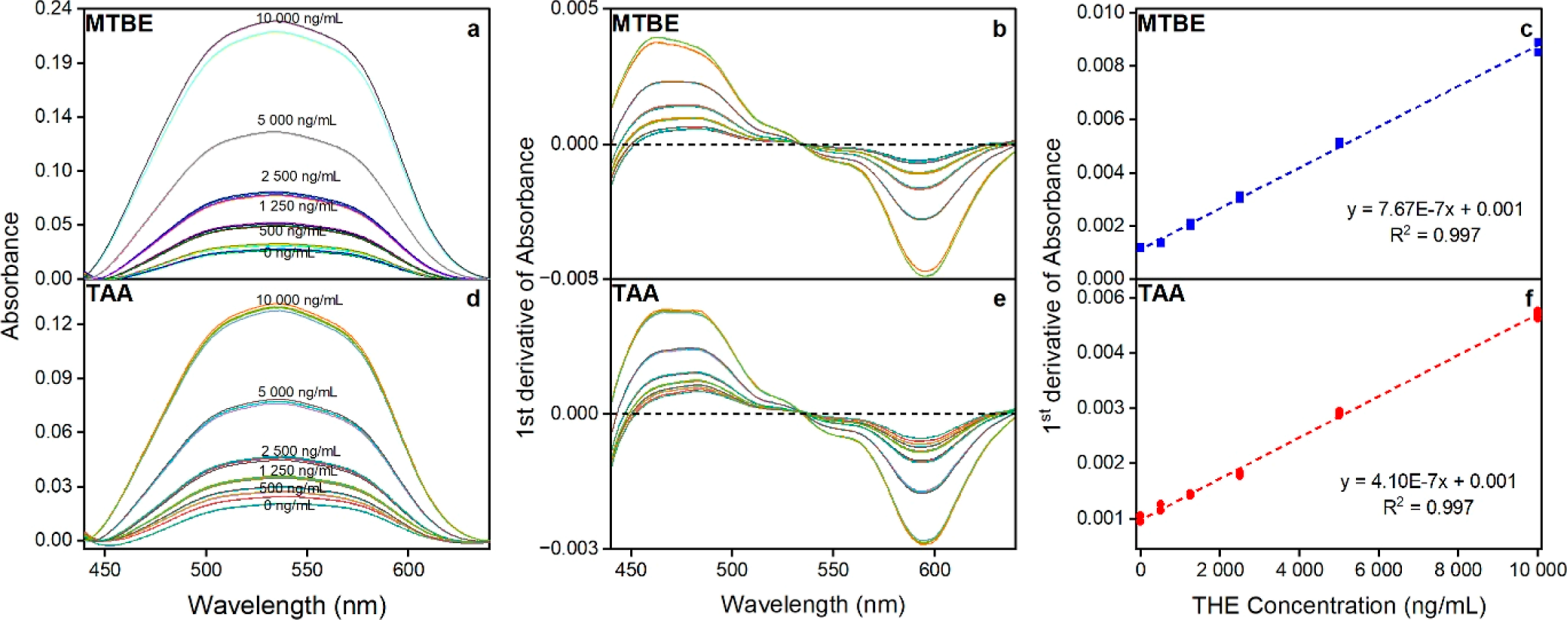
Spectra (a,d: baseline corrected raw data, b,e: first derivative) and calibration curves (c,f) for MTBE/MeOH (a–c) and TAA/water (d–f) systems.

However, the lowest detectable concentration (LOD) was higher than the expected 5β-THE concentration in hydrolyzed feces samples for both solvent systems (Table 4). This was also confirmed by the fact that no reaction was detected in the hydrolyzed samples when they were analyzed with the presented UV–vis methods. For this reason, no UV–vis data were obtained for the fecal samples collected for the present study. However, since the 5β-THE concentration in raw samples is above the LOD, we believe the method has a potential application if an appropriate sample preparation is in place. This will be the scope of a follow-up study in addition to a complete validation of the method.

**Table 4:** LOD and LOQ of the Method Depending on the Solvent System Used

	LOD, ng/mL (3.3σ/m)^a^	LOQ, ng/mL (10σ/m)^a^
MTBE/MeOH	394.75	1196.20
TAA/water	498.54	1510.72

	expected 5β-THE concentration in feces, ng/g^b^	
	Nonstressed fish	Stressed fish
raw sample	586.03	1944.17
hydrolyzed sample^c^	29.30	97.21

a LOD and LOQ calculation based on ICH Q2­(R2):^41^ σ (standard deviation) and m (slope) are obtained from linear regression.

b Based on the findings of Fanjara et al.^37^

c Sample diluted 20 times prior to hydrolysis.

## 3 Discussion

The consistency of low fecal 5β-THE levels measured during the monitoring period demonstrates the robustness, claimed by farmers, of the fish used in the present study. According to the fish farmers, these fish were particularly robust, with minor welfare issues and a low mortality rate of about 5% registered, despite the delousing they endured before slaughter. For comparison, in 2022, the average fish mortality rate was 16.6% at the national level and 20.7% in production area 5 (PO5), where the farm is located.^6^ The mortality rate was three times as high (above 15%) for the past 5 years both at the national level and in PO5.^6^ The increasing 5β-THE level toward the end of the sampling period may reflect the stress level experienced by the fish prior to amoebic gill disease (AGD) problems detected soon after the last sampling date. The AGD problems may have started during the sampling period but were not identified before late autumn 2022. Cao et al.^17^ reported similar observations of plasma and fecal cortisol increase prior to inflammatory/viral disease outbreaks in farmed Atlantic salmon. Studies have shown that the rising sea temperature in the summer and autumn months also triggers increasing stress levels and disease outbreaks in fish.^42–44^

The impacts of the delousing processes on fish were not captured in the 5β-THE measurement following the events. Although the 5β-THE level was slightly higher (but not significant, *p* = 0.06) on 3 July 2022, around 10 days after the first delousing, the level measured on 7 August, also around 10 days after the second delousing event, was relatively low. The fecal 5β-THE level most likely returned to the baseline within 10 days after the delousing. Meling et al.^22^ found that fecal cortisol in farmed Atlantic salmon postsmolt significantly dropped to a low level 8 days after sea transfer (considered a very stressful event for fish), compared to the level measured 4 days after the transfer. However, the difference in 5β-THE magnitude may be attributed to the difference in the temperature increase rate during the two periods. Prior to the first delousing, the sea temperature rose faster compared to the period preceding the second delousing event. Studies have shown that temperature is an important factor affecting fish welfare and mortality. The quick increase of this factor requires fast adaptation processes that can be stressful to fish.^43,44^ The high 5β-THE level after the first delousing may also be due to other events, such as net cleaning. However, this information was not available from the farmers. Cao et al.^45^ recently reported the observation of elevated fecal cortisol metabolites in farmed Atlantic salmon 20 h following net cleaning.

In the present study, the BT assay was chosen to simplify the analytical procedure for stress assessment in farmed Atlantic salmon. This assay and colorimetric assays in general present a cost-effective and rapid alternative to traditional techniques such as ELISA and LC–MS/MS for cortisol metabolite analysis. Compared to LC–MS/MS, which requires expensive instrumentation, specialized expertise, and extensive sample preparation,^24,25^ BT assays utilize a simple spectrophotometer, significantly reducing both costs and technical demands. While ELISA is less costly than LC–MS/MS and offers a moderate balance between affordability and sensitivity, it still relies on specific antibodies and plate readers. This dependency may introduce cross-reactivity issues and make it more resource-intensive than the BT assay. In terms of speed, the BT assay provides results within 10–20 min,^26,32^ making it considerably faster than ELISA, which requires 2–5 h, and LC–MS/MS, which, despite rapid analytical runs, is slowed by complex sample preparation. Additionally, the BT assay involves minimal sample processing, which further enhances its efficiency. However, these advantages come at the expense of their sensitivity and specificity. LC–MS/MS remains the most sensitive and specific method, capable of detecting cortisol metabolites at picograms per milliliter concentrations with precise differentiation between structurally similar compounds. ELISA, though less specific due to potential cross-reactivity, still offers higher sensitivity than the BT assay, detecting biomarkers in the pg/mL to ng/mL range.^8^ In contrast, the BT assay has a significantly higher detection limit (μg/mL or above) and is more susceptible to interference from other compounds. Despite these limitations, the BT assay’s simplicity, affordability, and speed make it particularly suitable for rapid, on-site applications where immediate stress assessment is more critical than exact quantification, particularly in aquaculture settings.

The results from the colorimetric method development in the present study are in accordance with those obtained by Graham et al.^46^ The authors found that tetrazolium formazan formation is inversely proportional to the dielectric constant of the solvent system used and directly proportional to the hydrogen-bonding capability of solvent mixtures with similar dielectric constant. Although the dielectric constants of the solvent mixtures used in the present study were not determined experimentally, values computed based on an empirical formula^47^ show that they increased with increasing percentage of MeOH or water. These solvents have higher dielectric constants than the primary solvents MTBE and TAA. According to the literature, MeOH has a high dielectric constant of 32.6 (at 20 °C)^48^ compared to MTBE (ε = 4.5 at 20 °C).^49,50^ Similarly, water has a high dielectric constant of 79.7 (at 20 °C)^51^ compared to TAA (ε = 5.82 at 25 °C).^52^ In the present study, 5β-THE absorbance decreased, and the time for reaching maximum absorbance increased when the fraction of high dielectric constant solvents (methanol or water) was increased. This effect was more pronounced for the TAA/water system than for the MTBE/MeOH system, as the dielectric constant range for the former was larger (ε ranging from 45.84 to 66.67) than for the latter (ε ranging from 20.86 to 25.07). Although slowing down the redox process, high dielectric constant solvents such as methanol and water still play an important role in the reaction process as they help stabilize the color development.^26^

High method sensitivity, as obtained by Ahmed,^32^ was desirable but not achieved in the present study. The authors managed to measure cortisol spiked with artificial saliva at very low concentrations (from 0.5 to 32.5 ng/mL). The discrepancies may be attributed to the difference in the corticosteroid entity analyzed (cortisol vs 5β-THE). According to Graham et al.,^53^ the reactivity of corticosteroids toward BT is partially affected by their molecular shape. The more planar the steroid molecule is, the faster its reaction with BT. The structure of 5β-THE differs from that of cortisol by the presence of a hydroxyl group (−OH) in C_3_, an alkane group (−CH) in C_5_, and a carbonyl group (C=O) in C_11_ (Figure 9). The first two functional groups make the A ring in 5β-THE less planar than that for cortisol and most likely affect its reactivity with BT negatively. However, Graham et al.^53^ also found that steroids having a carbonyl group in C_11_ react faster with BT than those lacking this functional group. Since 5β-THE possesses a carbonyl group instead of a hydroxyl group as in cortisol at C_11_, it is reasonable to assume that the reaction of 5β-THE to BT would not differ substantially from that of cortisol. This theory is also supported by the findings in the present study, being more or less in agreement with that obtained by Tu et al.^26^ The measurement range for cortisol obtained by these authors was also in the μg/mL level. Besides, no reaction was obtained within the indicated incubation time, with either cortisol or 5β-THE when we attempted to replicate the method described by Ahmed et al.^27^

**Figure 9:**
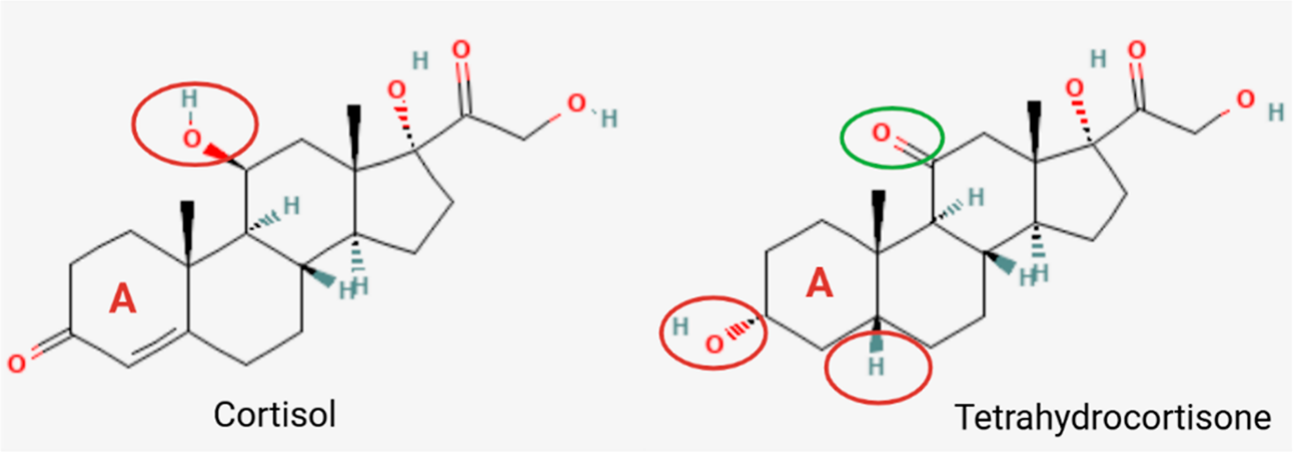
Comparison of cortisol and tetrahydrocortisone structures. The presence of the functional groups highlighted in red circles affects the compound’s reactivity to BT negatively, while the one in green circles has a positive effect, according to Graham et al.^53^ The compounds’ structure images were retrieved from PubChem (cortisol: https://pubchem.ncbi.nlm.nih.gov/compound/5754#section=2D-Structure and tetrahydrocortisone: https://pubchem.ncbi.nlm.nih.gov/compound/5866#section=2D-Structure).

Since the expected levels of 5β-THE in hydrolyzed feces samples (based on the LC–MS/MS data) were below the LOD of the presented colorimetric methods, no reaction was detected when measuring hydrolyzed feces samples. As a result, the development of a suitable sample treatment procedure is recommended before the method can be successfully implemented. Being able to perform analyses on site with a portable spectrophotometer is the aim of our studies and will be targeted when developing and optimizing the sample processing method. This implies that the method should be simple and easy to perform in an environment with limited availability of laboratory equipment. Unlike human sweat or saliva, which comprises 99% water,^26,27^ fish feces are a complex mixture of metabolites with varying polarities.^54^ Appropriate sample preparation and cortisol metabolites extraction are required before measurement to reduce interfering substances.^55^ Thus, the feasibility of analyzing fish skin mucus with the colorimetric method will also be investigated in addition to feces. This will reduce sampling invasiveness and simplify the stress evaluation of farmed fish. This will further increase the method’s suitability for use on-site. For instance, using the method based on the TAA/water system, a supernatant from centrifuged water-based skin mucus could be mixed with a methanolic solution of BT, followed by TAA and a methanolic solution of TMAOH before spectral acquisition with a portable spectrophotometer. This will reduce the time required for the analysis and be low-invasive for fish. Fish skin mucus was, however, found to contain a low amount of cortisol and requires a very sensitive method.^23,37^ Therefore, this was not considered in the present study. However, since measuring skin mucus will further reduce the invasiveness of the sampling procedure, as mentioned previously, assessing stress on-site using this matrix will be given particular attention and will be one of the focuses of our follow-up study.

Data quality can significantly affect the outcomes of the data analysis. Poor data may lead to inaccurate conclusions, misinformed decisions, and wasted resources. The analysis/modeling used here is a typical data-driven approach, indicating that the data quality is crucial and may strongly impact the achievements in the analysis. In the present work, we have experienced errors/inaccuracies that may be introduced when the instrument performs subtraction of the absorbance of the reagent blank. This was the case when the standard absorbance was read against a reagent blank during the method optimization. This procedure was necessary to eliminate the color development not attributed to 5β-THE.^56^ As described by Kleeman and Bailey,^57^ the color generated during the BT assay results from two reactions: the reaction of BT to corticosteroid (5β-THE in this case) and the formation of tetrazolium hydroxide, which rearranges to a colored isomer. Tetrazolium hydroxide arises from the unstable nature of BT in alkaline solutions and is an inevitable byproduct of the BT assay. This is, for instance, assumed to be the reason the color development accelerated when the TMAOH percentage was increased (increasing the pH) in the present study.

To minimize the error generated through reagent blank subtraction, it is recommended that the reagent blank solution be measured separately from the standard solution. Although another source of error may be introduced here as the reagent blank and standard are measured separately, it is assumed to be minimal compared to that obtained when the standard is measured against the reagent blank. This was not an issue when measuring samples with the same method (same experimental conditions) such as when measuring the calibration data. Although the background was also unstable, the differentiation procedure eliminated this problem. The use of replicates may also reduce the effects of varying instrumental backgrounds and can be used instead of separately measuring the standard and reagent blank separately.

Furthermore, using high-purity BT and solvents is also important for the BT assay. As Bush and Gale^58^ stated, the BT reagent’s impurity (presence of tetrazolium salts other than BT) may affect the results’ reproducibility. According to the authors, impurity causes irregular behavior, such as reversible light-induced color changes.^58^ Similarly, Sulkowitch et al.^59^ found a positive correlation between sample solvent impurity and color development in the reagent blank and emphasized the importance of checking this parameter. Based on the certificates of analyses provided by the manufacturers, the chemicals used in the present study were of great purity (99.7 to 100.5%; see Section 5.3.2). Thus, we assumed that the effect of BT or solvent impurities on the absorbance reading was minimal. However, observing slightly fluctuating absorption peaks during this study may suggest this issue. Different wavelengths for maximum absorbance were also reported for the cortisol BT assay in the literature: 510 nm,^26,27^ 525 nm,^46,53,56,57^ and 575 nm,^59^ which may be attributed to the difference in the solvents and experimental conditions used but could also be the difference in chemical purities.

Full validation of the methods (sample preparation and UV–vis detection) should be conducted following guidelines, such as those outlined in the ICH Q2­(R2), to determine their specificity/selectivity, accuracy and precision, range, and robustness.^41^ In addition to proper sample cleanup, the method’s specificity can be optimized using appropriate data treatment, such as higher-order derivative spectrophotometry.^60,61^ The work of Talsky et al.^60,62,63^ demonstrated, for instance, that higher-order derivatives (third, fourth, and fifth derivatives) could be helpful in situations where samples containing compounds with trace amounts or multiple compounds with superposed spectra are to be analyzed. Furthermore, multivariate analysis using multiwavelength absorbance data can also be used.^64^ This process requires, however, that a proper calibration model be first built by measuring an appropriate number of samples.

## 4 Conclusions

This paper reports the fecal 5β-THE levels measured in Atlantic salmon under normal farming conditions using LC–MS/MS. The data serve as reference values for future studies involving monitoring stress responses in farmed fish. Moreover, we introduce the potential to simplify the measurement of this cortisol metabolite (5β-THE) using a rapid colorimetric/UV–vis procedure based on the BT reaction. Low-toxicity solvents are used as primary solvents instead of methanol.

Despite its advances and increasing interest in human clinical research, no studies involving fish were found using this simple method at the time of writing this article. This is also the first time that the BT method was optimized for measuring 5β-THE instead of cortisol or other corticosteroids. These findings serve as a basis for further developing the method toward its application in real-life situations on fish farming sites. Measuring fish stress on-site offers an advantage as it allows us to understand the fish’s reactions to their environment and helps improve farm management and operations. Future research should optimize sample preparation and data treatment to enhance the method’s applicability. The availability of portable UV–vis spectrophotometers on the market makes this method attractive. It will allow on-site cortisol metabolite measurement, facilitating stress assessment in fish directly in aquaculture facilities without requiring extensive laboratory infrastructure and time to get valuable results.

## 5 Materials and Methods

### 5.1 Feces Sampling

The present study was conducted by collecting feces carefully stripped from 10 individual fish during routine sea lice counting events. Samples were collected 10 times over a period of about 6 months, from late February to early September 2022 (Figure 10).

**Figure 10:**
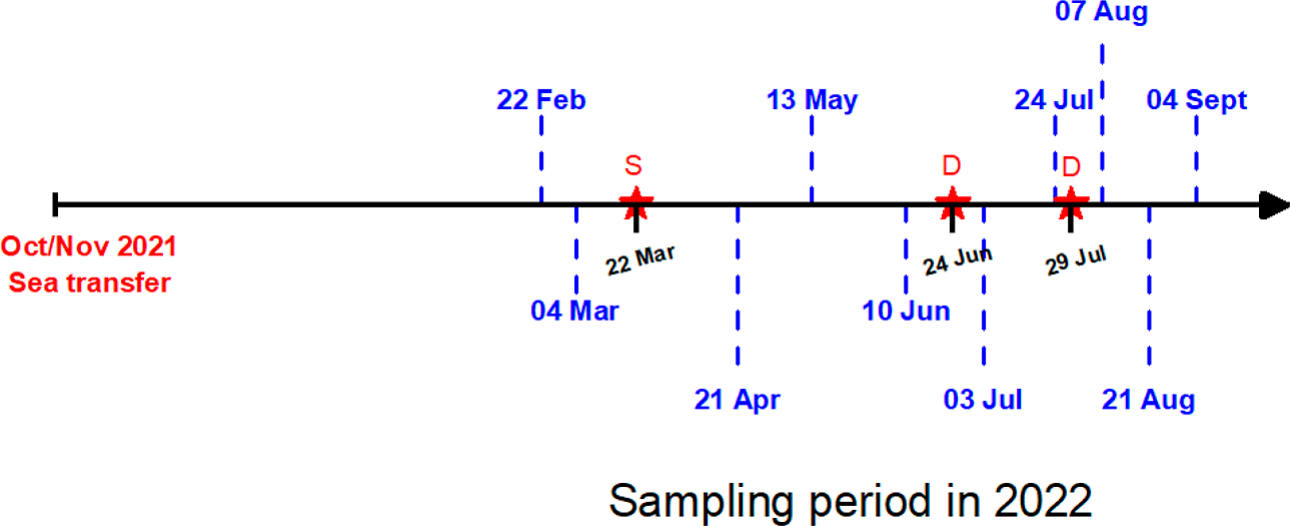
Timeline showing dates of feces sample collection. Red stars represent stress experiment (S) and fish delousing (D).

During this period, fish weighed on average between 453 and 2125 g. The fish population studied was transferred to sea in October/November 2021 and slaughtered in January 2023. Fish were reared at NTNU’s R&D license for farming salmonids, located in Aalesund municipality in Western Norway. The site is approved for research purposes by the Norwegian authorities. No further approval was needed as lice counting is a normal fish handling practice in salmon aquaculture.

### 5.2 LC–MS/MS Analysis of Fecal 5β-THE

Tetrahydrocortisone (5β-THE) was previously measured in fish feces using the LC–MS/MS method developed and validated by Fanjara et al.^23^ Details about the analytical method can be found in that work. Briefly, 100 mg of wet feces samples was first diluted 20 times, centrifuged, and 200 μL of supernatant hydrolyzed at 55 °C with β-glucuronidase from Helix pomatia for 1 h. Free cortisol metabolites were extracted with MTBE and recovered in 200 μL of 15% MeOH after drying the MTBE extract. The LC–MS/MS measurement was conducted using a gradient elution run for 15 min at a 0.3 mL/min flow rate. The analysis was performed in positive ionization mode and multiple reaction monitoring scanning. Samples were prepared and measured in duplicates. Analysis was performed using an Agilent 1200-series Ultra-performance liquid chromatography (Agilent, Santa Clara, California, USA) coupled with a Bruker Daltonics Compact Q-TOF mass spectrometer with ESI source (Billerica, Massachusetts, USA).^23^

Data on sea temperature, number of female sea lice per fish, delousing activity, and average monthly weight of fish during the sampling period were obtained from the farmers and BarentsWatch.no. No disease outbreak was registered during this period.

### 5.3 Colorimetric Method Development and Optimization

In order to simplify the measurements of fecal cortisol metabolite from farmed fish, a colorimetric method using BT as a reagent was first developed and optimized using a 5β-THE standard material before samples were analyzed with the method. The principle of the BT assay is as follows: in a strongly alkaline solution, BT oxidizes corticosteroids at the α-keto moiety of their C_17_ side chain and is reduced quantitatively to formazan.^46,53,57^ The concentration of the highly colored formazan can then be measured spectrophotometrically. The BT method presented by Tu et al.^26^ and Ahmed et al.^27,32^ for measuring cortisol in human sweat and saliva was used as a basis for the method development in the present study.

A BT methanolic solution was added to a standard solution of 5β-THE, followed by the addition of a TMAOH solution. The mixture was then incubated at room temperature, and a spectrum was acquired with a UV-1900i spectrophotometer from Shimadzu. Although the method was developed for cortisol measurement in the two studies cited above, it was optimized for measuring 5β-THE in the present study. In our previous studies, 5β-THE was determined as the predominant compound in feces with LC–MS/MS.^23,37^ Besides, cortisol and 5β-THE have similar compound structures and are expected to react with BT in similar ways and absorb in the same wavelength area. The BT method was previously demonstrated to be able to measure different types of corticosteroids in various sample matrices such as human saliva, sweat, urine, and pharmaceutical formulations.^56,57,59,65,66^

#### 5.3.1 Instrumentation

The analyses were conducted on a double-beam UV–vis spectrophotometer UV-1900i from Shimadzu (Filial Sweeden). Shimadzu’s LabSolutions UV–vis software version 1.14 was used for spectrum acquisition. Transparent UV cuvettes (H x W: 45 × 12.5 mm) with a 10 mm optical path length and 15 mm center height were purchased from Sarstedt (Nümbrech, Germany). The cuvettes are suitable for use from 220 nm and can be used for 50 μL to 2 mL work volumes.

#### 5.3.2 Chemicals and Reagents

BT chloride (BT, 100.5% purity) was purchased from Fisher Scientific Norway (Oslo, Norway). The TMAOH solution (TMAOH, 25.3% in methanol) and TAA (analytical grade, 99.7%) were purchased from Merck (Darmstadt, Germany). Methanol (MeOH, analytical grade, 100%) and MTBE (HPLC grade, 100%) were supplied by VWR International (Oslo, Norway). Ultrapure water was obtained from an Omnia water system equipped with an inline 0.2 μm filter (Stakpure, Niederahr, Germany). Tetrahydrocortisone standard (5β-THE, 98%) was purchased from Steraloids (Newport, Rhode Island, USA). A methanolic stock solution of 5β-THE was prepared by dissolving the purchased amount in an appropriate volume of methanol. The 5β-THE stock solution (166 600 ng/mL) was stored at −60 °C until further analysis.

#### 5.3.3 Experimental Screening Design and Optimization

Experimental designs provide a more efficient and correct analysis than the ″one-variable-at-a-time” (OVAT) approach, with other factors assigned fixed values. Unlike the OVAT approaches, suitable experimental designs can accommodate factor interactions. Screening designs are used to identify important factors during method optimization or robustness testing. Usually, two-level screening designs, such as fractional factorial and Plackett-Burman designs, are applied. In the present study, three predictor variables were considered, namely, BT concentration (reagent), TMAOH concentration (catalyst), and the percentage of solvent with high dielectric constant and hydrogen bonding capacity (Solvent B). These factors were selected based on the findings of published studies on the BT assay.^46,53,56,57^ Two response variables were considered separately: the maximum absorbance measured with a UV–vis spectrophotometer and the time to reach this maximum absorbance.

Furthermore, the stability of the maximum value over time was also assessed. Two sample solvent combinations were evaluated: MTBE with MeOH and TAA with water. MTBE and TAA are designated as Solvent A (primary solvent) and are solvents with a low dielectric constant. Methanol and water are defined as Solvent B (secondary solvent) and are solvents with high dielectric constants and hydrogen bonding capacity. These two solvent systems were chosen based on the solvent parameters determined by Graham et al.^46^ to be important in BT essays, namely, dielectric constant and hydrogen bonding capacity. Furthermore, MTBE and TAA were chosen due to their low toxicity compared to methanol, which was used as the primary solvent in the study of Tu et al.^26^ and Ahmed et al.^27^ In the present study, we used designs with 11 runs instead of 8. A 2 × 3 factorial design with eight runs only allows for discovering significant effects. Minor effects relative to the noise will be buried, and the effects plots may show nothing statistically significant. Figure 11 shows an overview of the experimental designs conducted.

**Figure 11:**
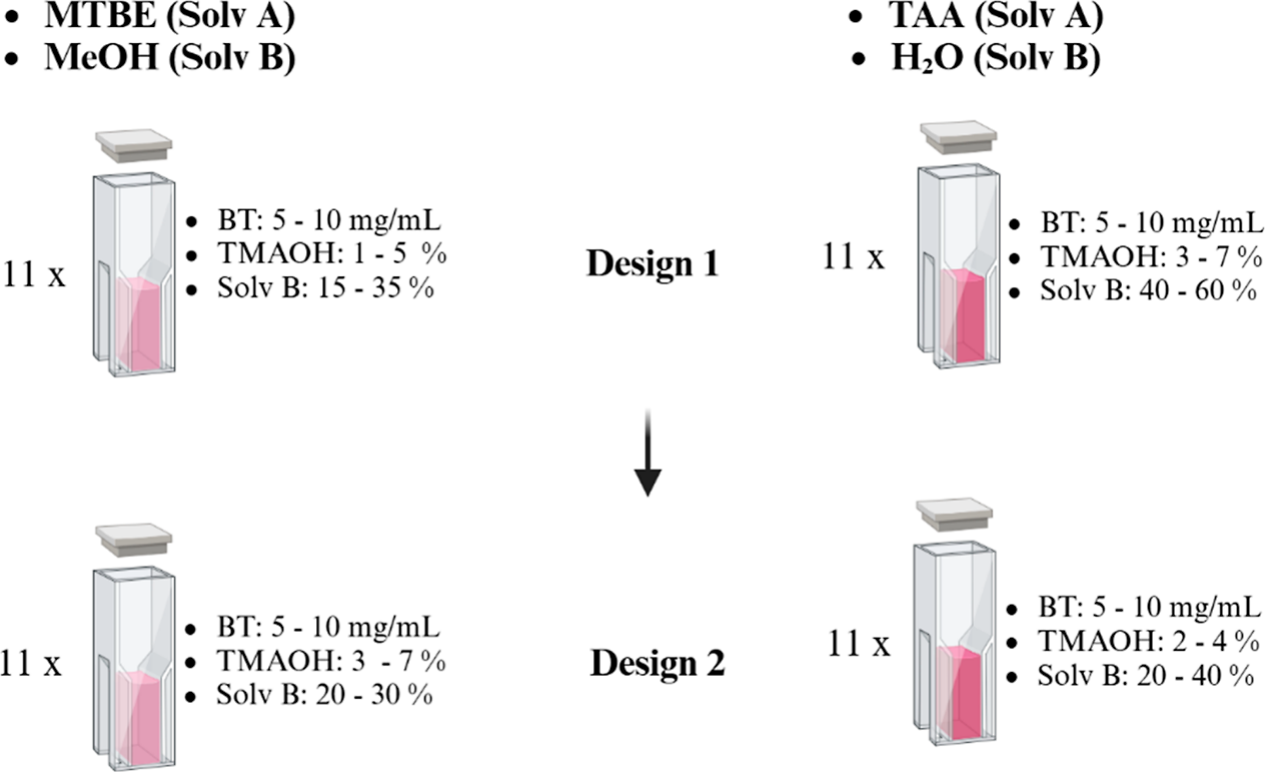
Overview of the experimental designs conducted for each solvent system. A complete design description is provided in Section 2.3. Created in https://BioRender.com.

Standard solutions of 10 000 ng/mL 5β-THE were prepared by diluting the stock solution with each solvent. The standard solution was prepared at the same concentration in each solvent to keep its concentration constant while varying the percentage of solvent B. This made a comparison of the spectra obtained from each run possible. Reagent solutions of 5, 7.5, and 10 mg/mL BT were prepared by dissolving 100 mg of the BT chloride powder in 10 mL of methanol, followed by appropriate dilution. Catalyst solutions of 1, 2, 3, 5, or 7% were prepared by diluting an appropriate volume of the 25% TMAOH solution with 5 mL of methanol. New solutions of the 5β-THE standard, reagent, and catalyst were prepared for each design.

For the screening/optimization procedure, two cuvettes were prepared for each run: one for the reagent blank (corticoid-free solvents) and one for the standard solution. For the latter, 25 μL of the BT solution was first added to the cuvette containing the 5β-THE standard solution in solvent B (Figure 12). Second, the 5β-THE standard in solvent A was added, followed by the addition of 25 μL of TMAOH solution. The solutions were then rapidly mixed with a vortex. A similar procedure was performed for the reagent blank cuvette, but a standard-free solvent and the same amounts of BT and TMAOH solutions were used. Both cuvettes were then transferred to their respective locations (sample and reference cuvettes) on the spectrophotometer. The spectrum was acquired against the reagent blank every minute for 18 to 20 min, and measurement was conducted at room temperature. Baseline correction of the instrument with empty cuvettes was performed prior to each batch of runs to minimize background and ambient noises. The spectrum was acquired from 350 to 700 nm, at a 1 nm data interval and a 1050 nm/min scan rate (scan speed: high speed).

**Figure 12:**
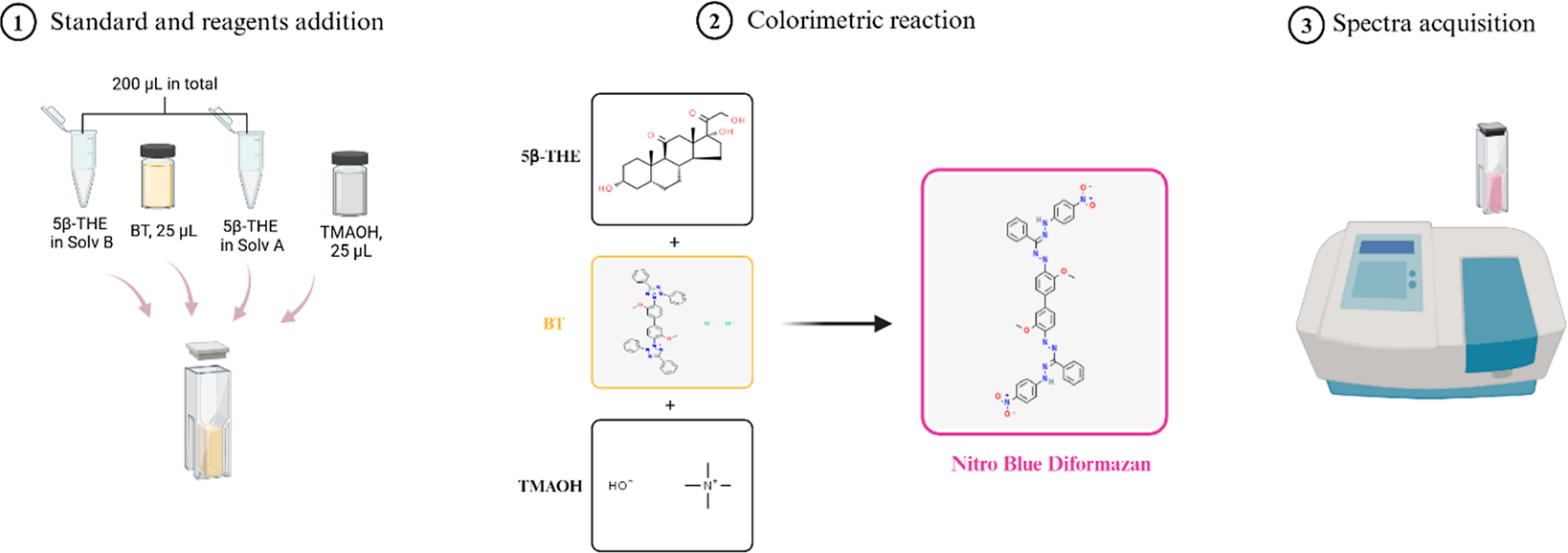
Simplified schematic of the BT reaction. Created in https://BioRender.com.

#### 5.3.4 Method’s Detection Limit and Fecal 5β-THE Measurement

A decreasing concentration of 5β-THE was measured to determine the lowest concentrations detectable by the method (LOD). The conditions determined during the experimental screening design and optimization procedure above were used for this purpose. Fecal samples were also analyzed using these conditions following the hydrolysis process explained in Section 5.2. Solvents (standard-free), reagents, and catalysts were added to the dry MTBE extracts in the same order as explained in Figure 12 before the solution was mixed and incubated at room temperature, and spectra were acquired.

### 5.4 Data Analysis

Model diagnostics were first performed for the fecal 5β-THE data from the LC–MS/MS measurement to detect outliers and check for the normality assumption using the Shapiro–Wilk test. Since the assumption was not met, data were Log_10_-transformed, and outliers were excluded to normalize the distribution before the one-way ANOVA test. Using Dunnett’s test, the means from each sampling point were compared to the mean of the control group from the stress experiment performed by Fanjara et al.^37^ The 5β-THE values for the control and 24 h groups from this study were used as reference values for nonstressed and stressed fish, respectively. The equality of variances of the groups was also checked through an ANOVA test. As suggested by Rosner, a nonparametric Kruskal–Wallis test was performed on the original, untransformed data and compared with the results from the ANOVA test to strengthen the conclusions obtained.^67^ All statistical analyses were carried out using IBM SPSS Statistics version 29.0.2.0 (20), and graphs were plotted using OriginPro 2024 version 10.1.0.170 (student version). Results were considered statistically significant when *p* < 0.05.

Sirius version 13.5 was used for design generation and regression analysis of the UV–vis data. First, the wavelength associated with maximum absorption was identified by using the raw spectra. This wavelength varied somewhat across the samples in the data set, and the most representative wavelength was selected. Furthermore, a scan was performed to investigate all recorded spectra from 0 to 20 min, identifying the spectra with the maximum absorption for all samples. The procedure ended with 11 spectra and 11 corresponding time values for the typical saturated design with three factors and three center points. These 11 UV–vis spectra were then smoothed with a moving average (window size 3) before first-order derivative data were generated (Savitsky-Golay smoothing, third order, and window size 11). The maximum absorbance value for each of the 11 runs from each design was obtained by subtracting the maximum and minimum values from the derived spectra. The maximum absorbance and the corresponding time were used as response variables in the analyses of the 2-factorial designs. Regression analysis using partial least squares was performed, and the resulting regression model was used to identify the significance of each of the predictor variables (BT, TMAOH, and percentage solvent B) and their interactions, using Lenth’s ME and simultaneous ME (SME).^40^ For a saturated design, no ANOVA information is available.

A plot of the regression coefficients is used to show the significance. Lenth’s ± ME and ± SME were used as reference limits in the regression plot. A factor with a regression coefficient exceeding the SME values was identified as significant, between SME and ME as uncertain, and below ME as nonsignificant. Since interpreting a regression model with strong interaction terms using only the regression coefficient plot is nearly impossible, a contour plot was used as this eases the interpretation.
